# P062 Treat inflammation or accept the damage? - “a diagnostic dilemma in advanced Takayasu arteritis”

**DOI:** 10.1093/rap/rkag113.092

**Published:** 2026-11-03

**Authors:** Saumya Dinithi Rupasinghe, Roger Bucknall

**Affiliations:** Robert Jones and Agnes Hunt Orthopedic Foundation Trust, Oswestry, United Kingdom; Robert Jones and Agnes Hunt Orthopedic Foundation Trust, Oswestry, United Kingdom

## Abstract

**Introduction:**

Takayasu arteritis (TAK) is a chronic granulomatous large-vessel vasculitis characterized by relapsing vascular inflammation that could progress into irreversible arterial stenosis and organ damage. The greatest challenge in clinical practice is to differentiate active diseases from permanent vascular injury, particularly in patients with longstanding diseases and multiple comorbidities. Advanced imaging does not necessarily correlate with disease activity, and the inflammatory indicators are often unreliable. Therefore, clinical assessment, imaging and patient-specific risk factors must be carefully integrated when making therapeutic decisions. We present a complicated case of longstanding Takayasu arteritis in which the decision of reconsidering biological therapy remained a challenge.

**Case description:**

An Asian-Bangladeshi woman with a more than 30-year history of Takayasu arteritis and no recent long-term immunosuppressive therapy was reviewed in our rheumatology service after a prolonged hiatus from follow-up. She could not recall her previous immunosuppressive treatment. Her disease course had been complicated by extensive irreversible vascular damage, including bilateral renal artery stenosis resulting in stage 4 chronic kidney disease, severe renovascular hypertension, recurrent pulmonary oedema, mesenteric arterial stenosis, diffuse aortic calcification, bilateral subclavian artery stenosis, and previous aortic root, ascending aorta, and hemiarch replacement for severe aortic regurgitation. MRI of the brain demonstrated multiple mature cerebral microhaemorrhages.

Left temporal artery tenderness and a carotid bruit were noted on examination. Raised inflammatory markers (ESR 70 mm/hour, CRP 20 mg/L), prompted treatment with high-dose prednisolone followed by subcutaneous tocilizumab as a steroid-sparing agent, with good symptomatic response and successful steroid tapering. Tocilizumab was discontinued after nine doses because of poorly controlled hypertension and overseas travel.

She subsequently developed worsening lower limb pain with persistently high inflammatory markers (ESR 88 mm/hour, CRP 34 mg/L). Involvement of great vessels, bilateral subclavian arteries, and severe bilateral renal artery stenosis were demonstrated in CT angiogram. Previous PET-CT had demonstrated vascular uptake suggestive of inflammation; however, interpretation was confounded by advanced irreversible vascular damage. Chronic kidney disease and anaemia further complicated interpretation of inflammatory markers.

This case was discussed at the multidisciplinary team meeting to determine whether her presentation represented active Takayasu arteritis or symptoms attributable predominantly to fixed vascular damage. The opinion was to carefully resume subcutaneous tocilizumab because it was impossible to definitively rule out persistent vascular inflammation. Owing to her history of recurrent infections and multiple comorbidities, treatment was resumed at an extended dosing interval of once every two months with close clinical, biochemical, and imaging surveillance.

**Discussion:**

Assessment of disease activity in longstanding Takayasu arteritis remains challenging as irreversible vascular damage and active inflammation frequently coexist. This patient demonstrated multiple adverse prognostic features including extensive aortic involvement, bilateral renal artery stenosis, mesenteric vascular disease, previous major vascular surgery and advanced chronic kidney disease. Consequently, neither symptoms nor inflammatory markers alone were sufficient to determine whether active vasculitis persisted.

Despite elevated ESR and CRP, these findings required cautious interpretation in the context of chronic kidney disease and chronic inflammation. Lower limb pain could reflect active vascular inflammation, chronic limb ischaemia or non-inflammatory causes. Cross-sectional imaging confirmed severe structural vascular damage but could not reliably differentiate active inflammation from established fibrosis. PET-CT offered additional functional assessment, although vascular uptake in longstanding disease may persist despite adequate immunosuppression and is not always synonymous with clinically significant disease activity.

The patient had previously shown a favourable clinical response to tocilizumab, allowing successful glucocorticoid taper. However, restarting biological therapy also carried significant risks, including infection, worsening renal complications and uncertainty regarding therapeutic benefit in the presence of extensive irreversible vascular injury. Conversely, failure to treat active inflammation risked further progression of vascular damage.

This case emphasizes the importance of multidisciplinary decision-making involving rheumatology, nephrology, vascular surgery and cardiology. Clinical evaluation, serial imaging, laboratory trends, and patient-centered discussions about the possible advantages and disadvantages of further immunosuppression had to be integrated into treatment decisions rather than depending on a single intervention.

The case raises an important question for discussion: when should clinicians recommence immunosuppression in patients with advanced Takayasu arteritis in whom irreversible vascular damage obscures assessment of ongoing disease activity?

**Key learning points:**

• Assessment of disease activity in advanced Takayasu arteritis requires careful distinction between active inflammation and irreversible vascular damage, as both could contribute to ongoing symptoms. In individuals with chronic renal disease, whose ESR and CRP may remain elevated regardless of vasculitis activity, relying solely on inflammatory markers is insufficient.

• Advanced imaging modalities provide complementary information. CT angiography accurately defines structural arterial damage, whereas PET-CT may identify metabolically active inflammation. However, both investigations have limitations and should always be interpreted within the wider clinical context rather than in isolation.

• This case illustrates the value of a multidisciplinary approach involving rheumatologists, nephrologists, vascular surgeons, cardiologists and radiologists when managing patients with complex large-vessel vasculitis. Collaborative assessment allows consideration of competing risks, including progression of vascular inflammation, cardiovascular complications, infection risk and treatment-related toxicity.

• Although Tocilizumab has emerged as a significant steroid sparing agent in Takayasu arteritis, question still surround regarding duration of treatment, indications for re-treatment following interruption and predictors of response in patients with advanced vascular damage. Current evidence offers limited guidance for patients with extensive established arterial disease, highlighting the need for individualised treatment decisions.

• This case also highlights how crucial it is to weigh the possible risks of immunosuppression against disease control. Restarting biological therapy might stop more inflammatory vascular damage, but it might not be very helpful in cases when irreversible structural illness is the primary cause of symptoms.

